# Prognostic role of inflammatory diets in colorectal cancer overall and in strata of tumor‐infiltrating lymphocyte levels

**DOI:** 10.1002/ctm2.1114

**Published:** 2022-11-27

**Authors:** Tomotaka Ugai, Li Liu, Fred K. Tabung, Tsuyoshi Hamada, Benjamin W. Langworthy, Naohiko Akimoto, Koichiro Haruki, Yasutoshi Takashima, Kazuo Okadome, Hidetaka Kawamura, Melissa Zhao, Seyed Mostafa Mousavi Kahaki, Jonathan N. Glickman, Jochen K. Lennerz, Xuehong Zhang, Andrew T. Chan, Charles S. Fuchs, Mingyang Song, Molin Wang, Kun‐Hsing Yu, Marios Giannakis, Jonathan A. Nowak, Jeffrey A. Meyerhardt, Kana Wu, Shuji Ogino, Edward L. Giovannucci

**Affiliations:** ^1^ Department of Pathology Program in MPE Molecular Pathological Epidemiology Brigham and Women's Hospital and Harvard Medical School Boston Massachusetts USA; ^2^ Department of Epidemiology Harvard T.H. Chan School of Public Health Boston Massachusetts USA; ^3^ Department of Epidemiology and Biostatistics, and the Ministry of Education Key Lab of Environment and Health, School of Public Health Huazhong University of Science and Technology Wuhan Hubei P. R. China; ^4^ Hubei Cancer Hospital Wuhan Hubei P.R. China; ^5^ Department of Nutrition Harvard T.H. Chan School of Public Health Boston Massachusetts USA; ^6^ Division of Medical Oncology, Department of Internal Medicine The Ohio State University College of Medicine and Comprehensive Cancer Center Columbus Ohio USA; ^7^ Department of Pathology, Beth Israel Deaconess Medical Center Harvard Medical School Boston Massachusetts USA; ^8^ Department of Pathology Center for Integrated Diagnostics Massachusetts General Hospital and Harvard Medical School Boston Massachusetts USA; ^9^ Channing Division of Network Medicine, Department of Medicine Brigham and Women's Hospital and Harvard Medical School Boston Massachusetts USA; ^10^ Clinical and Translational Epidemiology Unit Massachusetts General Hospital and Harvard Medical School Boston Massachusetts USA; ^11^ Division of Gastroenterology Massachusetts General Hospital Boston Massachusetts USA; ^12^ Department of Immunology and Infectious Diseases Harvard T.H. Chan School of Public Health Boston Massachusetts USA; ^13^ Genentech South San Francisco California USA; ^14^ Department of Biostatistics Harvard T.H. Chan School of Public Health Boston Massachusetts USA; ^15^ Department of Biomedical Informatics Harvard Medical School Boston Massachusetts USA; ^16^ Department of Medical Oncology Dana‐Farber Cancer Institute and Harvard Medical School Boston Massachusetts USA; ^17^ Broad Institute of MIT and Harvard Cambridge Massachusetts USA; ^18^ Department of Medicine Brigham and Women's Hospital and Harvard Medical School Boston Massachusetts USA; ^19^ Cancer Immunology Program Dana‐Farber / Harvard Cancer Center Boston Massachusetts USA

**Keywords:** adenocarcinoma, clinical outcome, colorectal neoplasm, immunology, precision medicine

## Abstract

**Background:**

Certain dietary patterns can elicit systemic and intestinal inflammatory responses, which may influence adaptive anti‐tumor immune responses and tumor behavior. We hypothesized that pro‐inflammatory diets might be associated with higher colorectal cancer mortality and that the association might be stronger for tumors with lower immune responses.

**Methods:**

We calculated an empirical dietary inflammatory pattern (EDIP) score in 2829 patients among 3988 incident rectal and colon carcinoma cases in the Nurses’ Health Study and Health Professionals Follow‐up Study. Using Cox proportional hazards regression analyses, we examined the prognostic association of EDIP scores and whether it might be modified by histopathologic immune reaction (in 1192 patients with available data).

**Results:**

Higher EDIP scores after colorectal cancer diagnosis were associated with worse survival, with multivariable‐adjusted hazard ratios (HRs) for the highest versus lowest tertile of 1.41 (95% confidence interval [CI]: 1.13–1.77; *P*_trend_ = 0.003) for 5‐year colorectal cancer‐specific mortality and 1.44 (95% CI, 1.19‐1.74; *P*_trend_ = 0.0004) for 5‐year all‐cause mortality. The association of post‐diagnosis EDIP scores with 5‐year colorectal cancer‐specific mortality differed by degrees of tumor‐infiltrating lymphocytes (TIL; *P*_interaction_ = .002) but not by three other lymphocytic reaction patterns. The multivariable‐adjusted, 5‐year colorectal cancer‐specific mortality HRs for the highest versus lowest EDIP tertile were 1.59 (95% CI: 1.01–2.53) in TIL‐absent/low cases and 0.48 (95% CI: 0.16–1.48) in TIL‐intermediate/high cases.

**Conclusions:**

Pro‐inflammatory diets after colorectal cancer diagnosis were associated with increased mortality, particularly in patients with absent or low TIL.

## Graphical abstract

1. Inflammation and adaptive immune responses play roles in the progression of colorectal cancer.

2. Higher intake of pro‐inflammatory diets after colorectal cancer diagnosis was associated with increased 5‐year mortality.

3. Prognostic association of pro‐inflammatory diets was stronger in tumors with fewer tumor‐infiltrating lymphocytes (TIL).

4. TIL serves as a predictive immunopathological biomarker for personalized adjuvant anti‐inflammatory diet interventions.

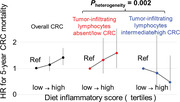

## 1 INTRODUCTION

Accumulating evidence indicates that inflammation plays a role in cancer growth, invasion and metastasis.^1^, ^2^, ^3^ Several inflammatory mediators, such as prostaglandins, IL6 (HGNC:6018; interleukin 6), CXCL8 (HGNC:6025; C‐X‐C motif chemokine ligand 8; aka, IL8), IL1A (HGNC:5991; interleukin 1 alpha) and TNF (HGNC:11892; tumor necrosis factor; aka, TNF‐alpha), have been reported to exert tumor‐promoting effects in colorectal cancer through activation of downstream signaling pathways, which may enhance angiogenesis and suppress the anti‐tumor immune response.^4^

Epidemiological and experimental evidence indicates that certain dietary patterns can influence local and systemic inflammatory status.^2^ An empirical dietary inflammatory pattern (EDIP) score has been developed as a surrogate metric of the summed inflammatory impact of various food items, using repeated dietary questionnaires and circulating inflammatory biomarkers from the same individuals.^5^ In the Nurses’ Health Study (NHS) and Health Professionals Follow‐up Study (HPFS), we showed a positive association of pro‐inflammatory diets with colorectal cancer incidence.^6^, ^7^ In addition, we also reported that pro‐inflammatory diets were associated with a higher risk of colorectal cancer subtypes with little or no peritumoral lymphocytic reaction,^6^ suggesting that such diets might contribute to the development of colorectal carcinoma through their suppressive effect on the adaptive anti‐tumour immune response.^8^ Another previous study has found that adherence to anti‐inflammatory diets might reduce total cancer mortality.^9^ However, the prognostic role of pro‐inflammatory diets after cancer diagnosis in specific cancer types remains uncertain. Diets after a cancer diagnosis are modifiable factors; therefore, it is of considerable importance to investigate which diets after the cancer diagnosis are beneficial in cancer patients.

Based on the aforementioned evidence, we hypothesized that pro‐inflammatory diets after colorectal cancer diagnosis might be associated with higher mortality, particularly in patients who had tumors with little immune response. To test our hypothesis, we investigated the prognostic association of post‐diagnosis EDIP scores in colorectal cancer patients overall and in strata of the intensity of the immune reaction to a tumor, utilizing data from two US‐based prospective cohort studies.

## 2 MATERIALS AND METHODS

### 2.1 Study population and ascertainment of colorectal cancer cases

**FIGURE 1:**
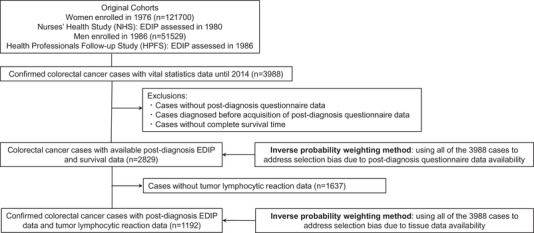
Flow diagram of the study population. Abbreviations: EDIP, Empirical Dietary Inflammatory Pattern; HPFS, Health Professionals Follow‐up Study; NHS, Nurses’ Health Study

Figure 1 illustrates an overall flow chart of study subjects. The NHS enrolled 121,700 registered female nurses residing in the United States aged 30–55 years at baseline in 1976, and the HPFS recruited 51,529 male health professionals residing in the United States aged 40–75 years at baseline in 1986.^10^, ^11^ Biennial questionnaires were sent to all study participants to collect and update demographic, lifestyle, medical and other health‐related data. To collect dietary information, validated food frequency questionnaires were administrated in 1980, 1984 and 1986, and every 4 years thereafter in the NHS, and 1986 and every 4 years thereafter in the HPFS. The study protocol was approved by the institutional review boards of Brigham and Women's Hospital and Harvard T.H. Chan School of Public Health, and those of participating registries as required.

In the two cohorts, when individuals reported a diagnosis of colon or rectal cancer in biennial questionnaires, we asked permission to acquire their medical records and pathological reports. Study physicians reviewed medical records to confirm the diagnosis of colorectal carcinoma and extracted relevant information on anatomic location, disease stage, and histopathological features. We identified deaths through the National Death Index and next‐of‐kin. When unreported lethal colorectal cancer cases were identified, we obtained consent from the next‐of‐kin to review medical records. All colorectal cancer deaths were confirmed by study physicians through a review of death certificates or medical records. Patients were followed until death, or December 2019 in both cohorts, whichever came first. We included cases diagnosed until 2014 to ensure adequate follow‐up periods (>5 years).

### 2.2 Assessment of the EDIP score and other covariables

The development and validation of the EDIP score were previously described in detail.^5^ The EDIP score is a weighted sum of 18 food groups that predicts concentrations of three plasma inflammatory biomarkers: IL6 (HGNC:6018), CRP (HGNC:2367; C‐reactive protein) and soluble TNFRSF1B (HGNC:11917; TNF receptor superfamily 1B; aka, TNFα‐receptor 2, TNFR2), following the standardized gene product nomenclature recommended by the expert panel.^12^ The food groups contributing to higher EDIP scores are the following: processed meat, red meat, organ meat, fish (other than dark‐meat fish), other vegetables (i.e. vegetables other than green‐leafy vegetables and dark‐yellow vegetables), refined grains, high‐energy beverages (cola and other carbonated beverages with sugar, fruit‐flavoured drinks), low‐energy beverages (low‐energy cola and other low‐energy carbonated beverages) tomatoes; whereas those contributing to lower EDIP scores are: beer, wine, tea, coffee, dark yellow vegetables (comprising carrots, yellow squash and sweet potatoes), green leafy vegetables, snacks, fruit juice and pizza. Higher (more positive) EDIP scores indicate pro‐inflammatory diets while lower (more negative) EDIP scores represent anti‐inflammatory diets.^5^ We calculated the EDIP score for each participant based on food frequency questionnaire data at each questionnaire cycle. To avoid the period of active anti‐cancer treatment, we used post‐diagnosis EDIP scores obtained through the first questionnaire returned between 6 and 48 months after colorectal cancer diagnosis in each patient, while pre‐diagnosis EDIP scores were obtained from the last questionnaire before colorectal cancer diagnosis. The latest available food questionnaire data of the NHS were in 2010, and those of the HPFS were in 2018.

Information on lifestyle and medical factors, including total energy intake, total physical activity, smoking, alcohol intake, endoscopy status, regular aspirin use, family history of first‐degree relative(s) with colorectal cancer, weight and height, was assessed using biennial questionnaires in both cohorts as previously described.^13^, ^14^

### 2.3 Assessment of histopathologic immune reaction and tumour characteristics

Attempts were made to collect available formalin‐fixed paraffin‐embedded tissue blocks of all incident colorectal carcinomas from hospitals where patients underwent tumor resection. The study pathologist (Shuji Ogino), who was unaware of other data, conducted a centralized pathology review of hematoxylin and eosin‐stained tissue sections to evaluate pathological features including tumor differentiation and four patterns of lymphocytic reaction (Crohn's‐like reaction, peritumoral reaction, intratumoral periglandular reaction and tumor‐infiltrating lymphocytes [TIL]).^15^, ^16^ Each lymphocytic reaction pattern was graded as absent/low, intermediate, or high, as previously described.^15^, ^16^ The second pathologist (Jonathan N. Glickman) independently re‐reviewed 398 selected cases, which showed good interobserver correlations, as previously described.^16^

We extracted DNA from the tumour and adjacent normal tissue, and assessed microsatellite instability (MSI) status, CpG island methylator phenotype (CIMP) status, mutation status for *BRAF* codon 600, *KRAS* codons 12, 13, 61 and 146, and *PIK3CA* exons 9 and 20, and long interspersed nucleotide element‐1 (LINE‐1) methylation level as previously described.^17^, ^18^, ^19^ We constructed tissue microarray for patients with sufficient tissue materials and detected the expression status of PTGS2 (HGNC:9605; aka, cyclooxygenase‐2) and CD274 (HGNC:17635; aka, PD‐L1) by immunohistochemistry.^20^, ^21^, ^22^

### 2.4 Statistical analysis

All statistical analyses were performed using SAS software (version 9.4; SAS Institute, Cary, NC). The two‐sided *p‐*value less than .005 was considered statistically significant as recommended by Benjamin et al.^23^ In our primary hypothesis testing, we examined the association of post‐diagnosis EDIP scores with colorectal cancer‐specific mortality. We also tested another primary hypothesis that the prognostic association of post‐diagnosis EDIP scores might differ by the histopathologic lymphocytic reaction. The primary outcome was colorectal cancer‐specific mortality, with all‐cause mortality considered as the secondary outcome. In the colorectal cancer‐specific mortality analysis, deaths from other causes were treated as censored events. In our primary hypothesis testing, we assessed a statistical trend for raw EDIP scores with cohort (sex)‐specific ceilings at 5th and 95th percentile values to eliminate outlier effects. In secondary analyses, EDIP scores were categorized into cohort‐specific tertiles. Survival time was defined as the time since colorectal cancer diagnosis to death or the end of follow‐up, whichever came first, accounting for left truncation because of variation between patients in the timing of post‐diagnosis EDIP assessment after diagnosis.^24^ Cumulative survival probabilities were estimated using the Kaplan‐Meier method and compared using the log‐rank test.

To evaluate the associations of post‐diagnosis EDIP scores with colorectal cancer‐specific and all‐cause mortality, we used Cox proportional hazard regression models stratified by sex and disease stage. The multivariable Cox regression models initially included age at diagnosis (continuous), year of diagnosis (continuous), tumor location (proximal colon vs. distal colon vs. rectum), tumor differentiation (well‐moderate vs. poor), family history of colorectal cancer (present vs. absent), pre‐diagnosis EDIP scores (continuous with cohort‐specific ceilings at 5th and 95th percentile values), post‐diagnosis aspirin use [regular use (≥2 tablets/week in the NHS or ≥2 times/week in the HPFS): (yes vs. no), cumulative pack‐years of smoking (continuous with a ceiling at 50 pack‐years), post‐diagnosis alcohol use (continuous with a ceiling at 30 g/day), post‐diagnosis physical activity (continuous with a ceiling at 50 metabolic equivalent task score‐hours/week), post‐diagnosis body mass index (continuous with a ceiling at 35 kg/m^2^) and post‐diagnosis total energy intake (continuous with cohort‐specific ceilings at 5th and 95th percentile values) as covariables. A backward stepwise elimination procedure with a threshold *p‐*value of .05 was used to select the variables for the final models. The inverse probability weighting (IPW) method^25^ was applied to reduce selection bias due to the availability of post‐diagnosis questionnaire data. The proportionality of the hazards assumption was assessed by a time‐varying covariate (i.e. the cross‐product of the post‐diagnosis EDIP score and survival time) and Schoenfeld residuals. The assumption was better satisfied for 5‐year survival analyses compared to analyses for the entire follow‐up period. Because most colorectal cancer‐specific deaths (68%) occurred during the first 5 years since diagnosis, we analyzed the 5‐year survival association with post‐diagnosis EDIP scores with censoring of all 5‐year survivors in our primary hypothesis testing.

To assess a statistical interaction between post‐diagnosis EDIP scores and histopathologic lymphocytic reaction, a *p*‐value for interaction (*P*_interaction_) was calculated using a Wald test for the cross‐product of post‐diagnosis EDIP score (continuous with the ceilings) and lymphocytic reaction (absent/low vs. intermediate/high; binary) in the multivariable‐adjusted Cox regression models. For tumor subtype and interaction analyses, the multivariable Cox regression models initially included the aforementioned covariates, MSI status (high vs. non‐high), CIMP status (high vs. low/negative), *KRAS* mutation (mutant vs. wild‐type), *BRAF* mutation (mutant vs. wild‐type), *PIK3CA* mutation (mutant vs. wild‐type), PTGS2 (cyclooxygenase‐2) expression (negative vs. positive) and LINE‐1 methylation level (continuous). A backward stepwise selection with *p‐*values of .05 was used to select variables for the final models. For tumor subtype analyses, the IPW method^25^ was applied to reduce selection bias due to the availability of tissue data. To avoid overfitting the models, the cases with missing data on categorical variables were included in the majority category. For cases with missing data on continuous variables, we substituted the mean value and assigned a separate indicator variable in the model.

## 3 RESULTS

During the follow‐up of 173,229 participants in the two prospective cohort studies (the NHS and HPFS cohorts), 3988 individuals had been diagnosed with colorectal carcinomas until 2014. Among these, 2829 cases had available post‐diagnosis EDIP data, and 1192 cases had data on post‐diagnosis EDIP scores and at least one component of histopathological immune reaction patterns (Figure 1). To adjust for selection bias, we used the IPW method and data from 3988 colorectal carcinoma cases to calculate the probability of availability of post‐diagnosis questionnaire data (or tissue data). The distribution of post‐diagnosis EDIP scores in each cohort is presented in Table S1. Post‐diagnosis EDIP scores were positively associated with body mass index and total energy intake and negatively associated with physical activity and total alcohol intake (Table 1 and Table S2 with tissue data available cases).

**TABLE 1:** Characteristics of patients with colorectal cancer according to tertiles of post‐diagnosis empirical dietary pattern scores in the Nurses’ Health Study (NHS) and the Health Professionals Follow‐up Study (HPFS)^¶^

		**Post‐diagnosis empirical dietary inflammatory pattern (EDIP) scores**			
**Characteristic** *	**All cases**	**Tertile 1 (lowest)**	**Tertile 2**	**Tertile 3 (highest)**	***p*‐Value** **^†^**
Participants (*n*)	2829	946	936	947	
Age at diagnosis, year	69.6 (8.9)	68.9 (8.8)	70.6 (8.8)	69.2 (9.1)	<.0001
Sex (n, %)					.82
Female (NHS)	1785 (63)	600 (63)	583 (62)	602 (64)	
Male (HPFS)	1044 (37)	346 (37)	353 (38)	345 (36)	
Year of diagnosis (*n*, %)					<.0001
Prior to 1995	915 (32)	335 (35)	253 (27)	327 (35)	
1996–2000	615 (22)	211 (22)	173 (18)	231 (24)	
2001–2014	1299 (46)	400 (42)	510 (54)	389 (41)	
Family history of colorectal cancer (*n*, %)	551 (19)	180 (19)	184 (20)	187 (20)	.91
Body mass index, kg/m^2^	25.9 (4.2)	25.3 (3.9)	26.0 (4.2)	26.4 (4.5)	<.0001
Alcohol intake, g/day	6.5 (9.3)	9.7 (10.5)	5.3 (8.2)	4.5 (8.1)	<.0001
Pack‐year of smoking	15.2 (18.0)	16.0 (17.9)	14.0 (17.3)	15.6 (18.7)	.045
Physical activity, METS ‐ h/week	14.8 (15.1)	17.4 (16.3)	14.0 (14.6)	13.1 (14.4)	<.0001
Regular aspirin user (*n*, %)	977 (37)	347 (38)	314 (38)	316 (35)	.34
Total energy intake, kcal/day	1795 (568)	1743 (568)	1735 (514)	1892 (589)	<.0001
Tumour location (*n*, %)					.26
Proximal colon	1180 (46)	383 (43)	393 (47)	404 (47)	
Distal colon	780 (30)	274 (31)	236 (29)	270 (31)	
Rectum	616 (24)	226 (26)	199 (24)	191 (22)	
Tumour differentiation (*n*, %)					.23
Well to moderate	1975 (84)	698 (85)	630 (83)	647 (82)	
Poor	389 (16)	121 (15)	127 (17)	141 (18)	
AJCC disease stage (*n*, %)					.001
I	710 (25)	269 (28)	218 (23)	223 (24)	
II	709 (25)	253 (27)	233 (25)	223 (24)	
III	608 (21)	207 (22)	194 (21)	207 (22)	
IV	186 (7)	51 (5)	59 (6)	76 (8)	
Unknown	616 (22)	166 (18)	232 (25)	218 (23)	
Tumour‐infiltrating lymphocytes (*n*, %)**					.76
Absent/low	860 (72)	313 (73)	258 (73)	289 (71)	
Intermediate/high	329 (28)	115 (27)	96 (27)	118 (29)	
Intratumoral periglandular reaction (*n*, %)**					.07
Absent/low	137 (12)	39 (9)	40 (11)	58 (14)	
Intermediate/high	1052 (88)	389 (91)	314 (89)	349 (86)	
Peritumoral reaction (*n*, %)**					.16
Absent/low	138 (12)	42 (10)	39 (11)	57 (14)	
Intermediate/high	1046 (88)	385 (90)	312 (89)	349 (86)	
Crohn's‐like reaction (*n*, %)**					.64
Absent/low	716 (73)	272 (75)	203 (72)	241 (72)	
Intermediate/high	265 (27)	92 (25)	80 (28)	93 (28)	

Abbreviations: AJCC, American Joint Committee on Cancer; EDIP, empirical dietary inflammatory pattern; HPFS, Health Professionals Follow‐up Study; METS, metabolic equivalent task score; NHS, Nurses’ Health Study.

^¶^ Post‐diagnosis empirical dietary inflammatory pattern scores were estimated based on the first questionnaire returned between 6 and 48 months after diagnosis of colorectal cancer.

* Continuous variables are shown as mean (standard deviation). Percentage (%) indicates the proportion of cases with a specific clinical, or pathological characteristic in all cases according to tertiles of post‐diagnosis empirical dietary inflammatory pattern scores.

^†^ To compare characteristics between subgroups, we used the chi‐square test for categorical variables and the analysis of variance for continuous variables.

** The numbers of colorectal cancer patients with data of tumor‐infiltrating lymphocytes, intratumoral periglandular reaction, peritumoral reaction, and Crohn's‐like reaction were 1186, 1186, 1181 and 978, respectively.

**FIGURE 2:**
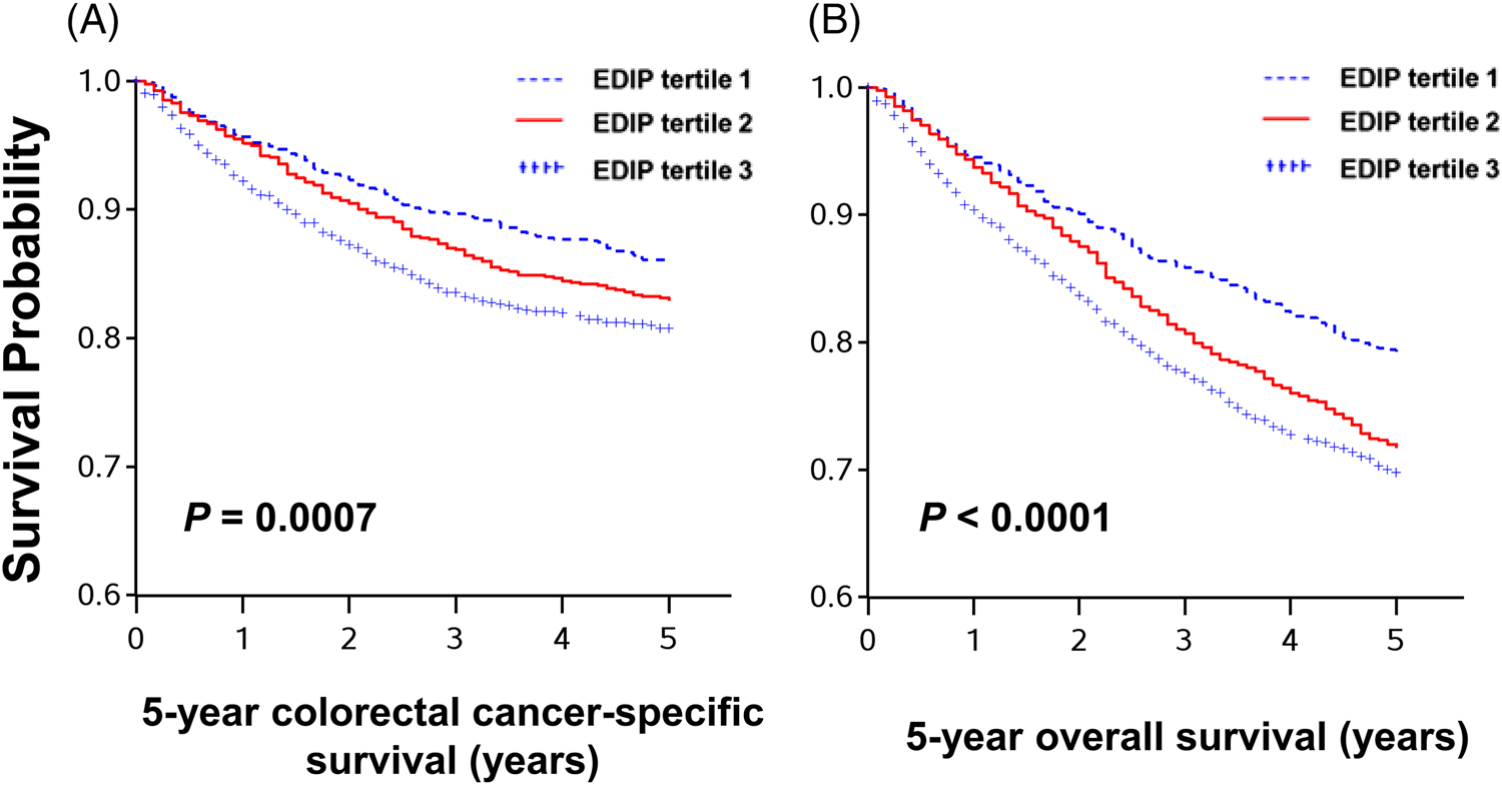
Kaplan‐Meier survival curves of patients with colorectal cancer according to post‐diagnosis empirical dietary inflammatory pattern (EDIP) scores among all confirmed colorectal cancer patients. The *p‐*values were calculated using the log‐rank test (two‐sided). A. 5‐year colorectal cancer‐specific survival; B. 5‐year overall survival

Among the 2829 colorectal carcinoma cases, there were 1829 all‐cause deaths, including 573 colorectal cancer‐specific deaths, during the median post‐diagnosis follow‐up time of 16.6 years for censored cases without 5‐year cutoffs. Since a vast majority of colorectal cancer‐specific deaths had occurred by 5 years after diagnosis and the survival curves showed separation of tertile categories of the post‐diagnosis EDIP score only up to 5 years of follow‐up (Figure 2 and Figure S1), we censored cases at 5 years from colorectal cancer diagnosis in further analyses.

In our primary hypothesis testing, higher post‐diagnosis EDIP scores were associated with increased 5‐year colorectal cancer‐specific mortality [multivariable‐adjusted hazard ratio (HR) for the highest versus lowest tertile, 1.41 (95% confidence interval [CI]: 1.13–1.77; *P*_trend_ = .003), and 5‐year all‐cause mortality (multivariable‐adjusted HR for the highest versus lowest tertile, 1.44 (95% CI: 1.19–1.74; *P*_trend_ = .0004; Table 2). Exploratory analyses by the components of EDIP scores showed an inverse association between coffee intake and a positive association of processed meat consumption with colorectal cancer mortality (Table S3).

**TABLE 2:** Post‐diagnosis empirical dietary inflammatory pattern scores and mortality among all confirmed colorectal cancer patients in the Nurses’ Health Study (NHS) and the Health Professionals Follow‐up Study (HPFS)^¶^

		**5‐year colorectal cancer‐specific mortality**			**5‐year all‐cause mortality**		
**Post‐diagnosis EDIP scores**	**No. of cases**	**No. of events**	**Age‐adjusted** **HR** * **(95% CI)**	**Multivariable HR** *, **^†^** **(95% CI)**	**No. of events**	**Age‐adjusted** **HR** * **(95% CI)**	**Multivariable** **HR** *, **^†^** **(95% CI)**
Tertile 1	946	103	1 (reference)	1 (reference)	148	1 (reference)	1 (reference)
Tertile 2	936	129	1.14 (0.90–1.44)	1.14 (0.90–1.45)	201	1.22 (1.01–1.49)	1.18 (0.97–1.44)
Tertile 3	947	160	1.45 (1.15–1.81)	1.41 (1.13–1.77)	237	1.52 (1.25‐1.84)	1.44 (1.19–1.74)
*P*_trend_ **			.002	.003		<.0001	.0004

Abbreviations: CI, confidence interval; EDIP, empirical dietary inflammatory pattern; HR, hazard ratio.

^¶^ Post‐diagnosis EDIP scores were estimated based on the first questionnaires returned between 6 and 48 months after diagnosis of colorectal cancer.

* The inverse probability weighting method (for post‐diagnosis questionnaire data availability) was integrated into the Cox proportional hazards regression models. All Cox regression models were stratified by sex and disease stage and adjusted for age at diagnosis.

^†^ Multivariable Cox regression models originally included the following variables: year of diagnosis, tumor differentiation, tumor location, family history of colorectal cancer, pre‐diagnosis empirical dietary pattern scores, post‐diagnosis aspirin use, post‐diagnosis pack‐years of smoking, post‐diagnosis alcohol use, post‐diagnosis physical activity, post‐diagnosis body mass index, and post‐diagnosis total energy intake. A backward stepwise selection was used to select the variables for the final models.

** *P*_trend_ was calculated using the EDIP score as a continuous variable with the cohort‐specific ceilings of the 5th and 95th percentiles.

Although the interaction was not statistically significant, stratified analyses by body mass index indicated a stronger association between post‐diagnosis EDIP scores and colorectal cancer‐specific mortality in overweight/obese cases (multivariable‐adjusted 5‐year colorectal cancer‐specific mortality HR for the highest versus lowest tertile, 2.13 (95% CI: 1.34–3.38) than normal weight cases (multivariable‐adjusted 5‐year colorectal cancer‐specific mortality HR for the highest versus lowest tertile, 1.10 (95% CI: 0.80–1.51; *P*_interaction_ = .066; Table S4). Stratified analyses by disease stage indicated that the prognostic associations of post‐diagnosis EDIP score were observed regardless of the stage (*P*_interaction_ = .33 for 5‐year colorectal cancer‐specific mortality; Table S5). A sensitivity analysis including all identified deaths until the overall study end date demonstrated a similar association for all‐cause mortality and an attenuated association for colorectal cancer‐specific mortality (Table S6). To minimize the effect of anti‐cancer treatment, we conducted another sensitivity analysis by utilizing EDIP scores derived from the first questionnaire returned between 12 and 48 months after diagnosis, which revealed similar prognostic associations of EDIP scores (Table S7). Furthermore, we conducted another sensitivity analysis adjusting for the time from cancer diagnosis to the timing of post‐diagnosis EDIP assessment after cancer diagnosis and observed similar findings (Table S8).

In the 1192 cases with available immune reaction data, we identified 218 all‐cause deaths, including 144 colorectal cancer‐specific deaths, up to 5 years after the cancer diagnosis. The association between post‐diagnosis EDIP scores and 5‐year colorectal cancer‐specific mortality differed by the intensity of TIL (*P*_interaction_ = .002; Table 3) but not by intratumoral periglandular reaction, peritumoral reaction, or Crohn's‐like reaction. The multivariable‐adjusted 5‐year colorectal cancer‐specific mortality HRs for the highest versus lowest tertile of post‐diagnosis EDIP scores were 1.59 (95% CI: 1.01–2.53) in TIL‐absent/low cases and 0.48 (95% CI: 0.16–1.48) in TIL‐intermediate/high cases.

**TABLE 3:** Post‐diagnosis empirical dietary inflammatory pattern scores and 5‐year mortality in strata of cases based on the lymphocytic reaction in colorectal cancer in the Nurses’ Health Study (NHS) and the Health Professionals Follow‐up Study (HPFS)^¶^

			**5‐year colorectal cancer‐specific mortality**			**5‐year overall mortality**		
**Strata of cases**	**Post‐diagnosis EDIP scores**	**No. of cases**	**No. of events**	**Age‐adjusted** **HR** * **(95% CI)**	**Multivariable** **HR** *, **^†^** **(95% CI)**	**No. of events**	**Age‐adjusted** **HR** * **(95% CI)**	**Multivariable** **HR** *, **^†^** **(95% CI)**
**Tumor‐infiltrating lymphocytes**								
Absent/low	Tertile 1	313	28	1 (reference)	1 (reference)	48	1 (reference)	1 (reference)
	Tertile 2	258	39	1.23 (0.74–2.02)	1.32 (0.81–2.15)	50	1.02 (0.67–1.55)	1.09 (0.72–1.64)
	Tertile 3	289	50	1.56 (0.98–2.49)	1.59 (1.01–2.53)	76	1.55 (1.06–2.25)	1.53 (1.05–2.23)
Intermediate/high	Tertile 1	115	12	1 (reference)	1 (reference)	15	1 (reference)	1 (reference)
	Tertile 2	96	8	1.04 (0.40–2.70)	0.83 (0.31–2.26)	12	1.04 (0.46–2.34)	0.88 (0.39–2.01)
	Tertile 3	118	6	0.62 (0.21–1.78)	0.48 (0.16–1.48)	15	1.07 (0.49–2.34)	0.91 (0.41–2.02)
*P*_interaction_ ^‡^				.012	.002		.15	.10
**Intratumoral periglandular reaction**								
Absent/low	Tertile 1	39	3	1 (reference)	1 (reference)	8	1 (reference)	1 (reference)
	Tertile 2	40	10	4.38 (1.14–16.8)	3.93 (1.07–14.5)	10	1.78 (0.66–4.78)	1.70 (0.66–4.39)
	Tertile 3	58	13	3.47 (0.93–12.9)	2.96 (0.81–10.9)	18	1.86 (0.75–4.62)	1.54 (0.61–3.89)
Intermediate/high	Tertile 1	389	37	1 (reference)	1 (reference)	55	1 (reference)	1 (reference)
	Tertile 2	314	37	0.97 (0.60–1.57)	0.99 (0.62–1.59)	52	0.94 (0.63–1.41)	0.95 (0.64–1.42)
	Tertile 3	349	43	1.16 (0.74–1.82)	1.16 (0.74–1.83)	74	1.37 (0.95–1.98)	1.34 (0.93–1.94)
*P*_interaction_ ^‡^				.17	.11		.54	.57
**Peritumoral reaction**								
Absent/low	Tertile 1	42	3	1 (reference)	1 (reference)	9	1 (reference)	1 (reference)
	Tertile 2	39	11	3.49 (0.93–13.0)	3.34 (0.93–12.0)	11	1.56 (0.62–3.90)	1.53 (0.63–3.70)
	Tertile 3	57	13	2.44 (0.65–9.15)	2.67 (0.75–9.49)	17	1.35 (0.56–3.26)	1.34 (0.57–3.16)
Intermediate/high	Tertile 1	385	37	1 (reference)	1 (reference)	54	1 (reference)	1 (reference)
	Tertile 2	312	36	0.98 (0.61–1.59)	1.00 (0.62–1.61)	51	0.96 (0.64–1.44)	0.97 (0.65–1.45)
	Tertile 3	349	42	1.17 (0.74–1.84)	1.13 (0.71–1.80)	74	1.41 (0.98–2.05)	1.35 (0.93–1.96)
*P*_interaction_ ^‡^				.39	.13		.94	.76
**Crohn's‐like reaction**								
Absent/low	Tertile 1	272	26	1 (reference)	1 (reference)	44	1 (reference)	1 (reference)
	Tertile 2	203	36	1.42 (0.84–2.38)	1.44 (0.87‐2.38)	44	1.14 (0.74–1.77)	1.20 (0.78–1.84)
	Tertile 3	241	39	1.38 (0.82–2.31)	1.25 (0.74‐2.09)	59	1.39 (0.92–2.09)	1.29 (0.85–1.96)
Intermediate/high	Tertile 1	92	9	1 (reference)	1 (reference)	12	1 (reference)	1 (reference)
	Tertile 2	80	6	0.81 (0.28–2.33)	0.73 (0.25‐2.09)	11	0.98 (0.42–2.28)	0.88 (0.38–2.04)
	Tertile 3	93	5	0.63 (0.21–1.91)	0.60 (0.21–1.72)	13	1.02 (0.45–2.31)	0.96 (0.43–2.14)
*P*_interaction_ ^‡^				.37	.36		.59	.61

Abbreviations: CI, confidence interval; EDIP, empirical dietary inflammatory pattern; HR, hazard ratio.

^¶^ Post‐diagnosis EDIP scores were estimated based on the first questionnaire returned between 6 and 48 months after diagnosis.

* The inverse probability weighting method (for tumor tissue data availability) was integrated into the Cox proportional hazards regression models. All Cox regression models were stratified by age and disease stage and adjusted for age at diagnosis.

^†^ Multivariable Cox regression models originally included the following variables: year of diagnosis, tumor differentiation, tumor location, microsatellite instability, CpG islands methylator phenotype, BRAF mutation, KRAS mutation, PTGS2 (cyclooxygenase 2) expression, PIK3CA mutation, LINE‐1 hypomethylation, family history of colorectal cancer, pre‐diagnosis empirical dietary pattern scores, post‐diagnosis aspirin use, post‐diagnosis pack‐years of smoking, post‐diagnosis alcohol use, post‐diagnosis physical activity, post‐diagnosis body mass, and post‐diagnosis total energy intake. A backward stepwise selection was used to select the variables for the final models.

^‡^ Pinteraction (two‐sided) was calculated by the Wald test for the cross‐product of post‐diagnosis EDIP scores (continuous with ceilings at 5th and 95th percentiles) and each lymphocytic reaction component (binary) in the Cox regression model.

## 4 DISCUSSION

Based on data from two large prospective cohort studies, we found that more pro‐inflammatory diets indicated by higher EDIP scores after colorectal cancer diagnosis were associated with increased 5‐year mortality. Further analyses by strata of immune responses showed that the positive association between post‐diagnosis EDIP scores and colorectal cancer‐specific mortality was observed in patients with absent/low TIL, but not in patients with intermediate/high TIL. Although validation in independent datasets is needed, our findings provide the first population‐based evidence for the modifying role of the colorectal tumor immune microenvironment in the adverse prognostic association of inflammatory diets.

In the NHS and HPFS, we previously found that an empirically identified pro‐inflammatory dietary pattern, characterized in part by foods high in sugar‐sweetened beverages, red and processed meat and low in green‐leafy and dark‐yellow vegetable intake,^5^ was associated with a higher risk incidence of colorectal cancer incidence in both men and women.^6^, ^7^ Other studies also found an association between pro‐inflammatory diets and cancer mortality in overall populations.^9^, ^26^, ^27^ The current study that further explored the prognostic value of the pro‐inflammatory dietary pattern information specifically in colorectal cancer patients indicates an adverse prognostic association of post‐diagnosis pro‐inflammatory diets. Two previous studies examined the association between a post‐diagnosis dietary inflammatory index (DII) and mortality in colorectal cancer patients.^28^, ^29^ Both of them used a nutrient‐based DII mainly driven by nutritional supplements. Ratjen et al. examined the association between post‐diagnosis DII scores and all‐cause mortality in long‐term survivors of colorectal cancer and found an adverse prognostic association of post‐diagnosis DII in patients with metastatic disease at diagnosis but not in the overall study population during a median follow‐up time of 7 years.^28^ Due to limited data, the study^28^ examined all‐cause mortality but did not examine colorectal cancer‐specific mortality. Zheng et al. calculated DII scores from food and supplements and found that the lowest DII score tertile was associated with lower all‐cause mortality (for the lowest vs. highest tertile; multivariable HR of 0.49 (95% CI: 0.31–0.79).^29^ Another study has shown that post‐diagnosis pro‐inflammatory diets are associated with an increased risk of cancer recurrence and mortality among breast cancer patients.^30^ Considering previous evidence and our findings, pro‐inflammatory diets after colorectal cancer diagnosis likely have an adverse effect on cancer survival.

Our study showed that the association between pro‐inflammatory diets and colorectal cancer‐specific mortality was even higher among overweight or obese colorectal cancer patients, which parallels the stronger association between EDIP and inflammatory markers in overweight/obese individuals.^7^ Moreover, the EDIP had much stronger associations with adverse metabolomic patterns in overweight compared to normal‐weight women.^31^ Obesity is linked to subclinical inflammation through induction of multiple pro‐inflammatory factors, such as IL1A, IL6, CXCL8 (IL8) and TNF.^32^, ^33^ Short‐chain fatty acids (by‐products of fermentation of dietary fiber), carotenoids and flavonoids (both enriched in yellow, orange and red vegetables) were reported to reduce levels of these pro‐inflammatory cytokines and may influence the secretion of anti‐inflammatory mediators.^34^, ^35^ The increase of pro‐inflammatory mediators or decrease of anti‐inflammatory mediators may activate transcription factors that facilitate the progression of tumors.^4^

Accumulating evidence suggests that gut inflammation causes impaired mucosal barriers and dysfunction of the immune system, which may lead to cancer development and progression.^36^, ^37^ In support, we found that the positive association between post‐diagnosis EDIP scores and colorectal cancer‐specific mortality was only observed in patients with absent/low TIL. Our previous study showed that pro‐inflammatory diets based on EDIP scores were associated with a higher incidence of the colorectal cancer subtype with low/no peritumoral reaction (HR for highest versus lowest EDIP score quintile, 2.60 (95% CI, 1.60–4.23; *P*_trend_ < .001) but not with the intermediate/high peritumoral reaction (*P*_trend_ > .80).^6^ Clinical outcomes of patients after tumor resection are substantially influenced by the presence (or absence) of residual tumor cells that are undetectable at the time of tumor resection (i.e. micrometastases). Residual tumor cells likely have characteristics and antigenicity for host immune cells similar to those of resected tumor cells. This study suggests that unhealthy diets with high EDIP scores may help those residual tumor cells proliferate after tumor resection, thereby increasing mortality. Based on our findings, clinicians might consider recommending a dietary pattern to limit inflammation as a potential strategy to improve survival in colorectal cancer patients with absent/low lymphocytic reaction, which has been shown to be a clinically more aggressive cancer subtype.^15^

Our study has several limitations. First, the diet data were self‐reported and had measurement errors to some extent. Yet, prior studies in these two cohorts have shown reasonably a good correlation between food frequency questionnaire (FFQ)‐derived data and data from diet records, suggesting that dietary intake can be measured by FFQ with similar validity as diet records.^5^ Moreover, measurement errors typically attenuate associations. Second, information on cancer treatment was limited. However, about 60% of our patients had stage I or II diseases, for which surgery without chemotherapy was the standard treatment. Given that all participants were health professionals, we would expect that a high proportion of stage III or above cancer patients received standard therapy. Third, although the EDIP was empirically constructed to assess the inflammatory potential of diet, it is correlated with other potentially adverse aspects of diet and lifestyle. However, we adjusted for a variety of potential confounders to improve the validity of our findings.

One major strength of this study is the prospective design and the integration of biennially prospectively collected data on diet and lifestyle exposures combined with tumoral features related to the host's immune response, allowing us to examine the prognostic associations of post‐diagnosis pro‐inflammatory diets stratified by histopathological immune response while adjusting for a variety of potential confounders. This design is an application of the molecular pathological epidemiology approach, which can provide novel insights into the effect of exposures on disease outcomes and identify patient subgroups associated with benefits from dietary/lifestyle modifications, thereby contributing to precision medicine.^38^, ^39^, ^40^ The prospective design also enabled us to utilize information from the cancer cases without postdiagnosis questionnaire data or tumoral tissue data and adjust for selection bias in the cases with both dietary and tumour tissue data by means of the IPW method. Furthermore, the availability of pre‐diagnosis dietary data enabled the adjustment for pre‐diagnosis EDIP scores to assess the independent association of post‐diagnosis pro‐inflammatory diet intake on colorectal cancer survival. Importantly, our colorectal cancer cases were derived from a large number of hospitals throughout the US, increasing the generalizability of our findings. However, our results need to be validated in independent datasets.

In conclusion, we found that pro‐inflammatory diets after colorectal cancer diagnosis were associated with increased mortality, particularly among patients who have tumours with absent or low TIL. Our findings, if validated, may have clinical implications for personalized adjuvant anti‐inflammatory diet interventions in the era of precision medicine.

## CONFLICT OF INTEREST

C.S.F. is currently employed by Genentech/Roche and has served as a consultant for Lilly, Sanofi, Bayer, Celgene, Merck, Bristol‐Myers Squibb, Entrinsic Health, Five Prime Therapeutics and Agios. M.G. receives research funding from Servier and Janssen. J.A.M. has received institutional research funding from Boston Biomedical, has served as an advisor/consultant to Ignyta and COTA Healthcare, and served on a grant review panel for the National Comprehensive Cancer Network funded by Taiho Pharmaceutical. K.‐H.Y. is an inventor of US Patent 10,832,406 (not related to this study). This study was not funded by any of these companies. The other authors declare that they have no conflicts of interest.

## FUNDING INFORMATION

This work was supported by US National Institutes of Health (NIH) grants (P01 CA87969 to M.J. Stampfer; UM1 CA186107 to M.J. Stampfer; P01 CA55075 to W.C. Willett; UM1 CA167552 to W.C. Willett; U01 CA167552 to W.C. Willett and L.A. Mucci; R01 CA137178 to A.T.C.; K24 DK098311 to A.T.C.; R35 CA197735 to S.O.; R01 CA151993 to S.O.; R03 CA197879 to K.W.; R21 CA222940 to K.W.; R21 CA230873 to K.W. and S.O.; K07 CA188126 to X.Z.; R37 CA225655 to J.K.L.; R35 GM142879 to K.‐H.Y.); by Cancer Research UK Grand Challenge Award (C10674 / A27140, to M.G. and S.O.); by Nodal Award (2016‐02) from the Dana‐Farber / Harvard Cancer Center (to S.O.); by an Investigator Initiated Grant from the American Institute for Cancer Research (AICR) (to K.W.); by the Stand Up to Cancer Colorectal Cancer Dream Team Translational Research Grant (SU2C‐AACR‐DT22‐17 to C.S.F. and M.G.), administered by the American Association for Cancer Research, a scientific partner of SU2C; and by grants from the Project P Fund, Bennett Family Fund and the Entertainment Industry Foundation through National Colorectal Cancer Research Alliance. J.A.M. is partially supported by the Douglas Gray Woodruff Chair fund, the Guo Shu Shi Fund, Anonymous Family Fund for Innovations in Colorectal Cancer, the P Project and the George Stone Family Foundation. L.L. was supported by a grant from the National Natural Science Foundation of China No. 81302491, a scholarship grant from the Chinese Scholarship Council and a fellowship grant from Huazhong University of Science and Technology. H.K. was supported by a fellowship grant from the Uehara Memorial Foundation. T.U. was supported by grants from the Japan Society for the Promotion of Science (201960541), Mishima Kaiun Memorial Foundation and Prevent Cancer Foundation. The content is solely the responsibility of the authors and does not necessarily represent the official views of NIH. The funders had no role in study design, data collection and analysis, decision to publish, or preparation of the manuscript.

## Supporting information

Supporting Information
