# Supplementary material for: Prognostic role of inflammatory diets in colorectal cancer overall and in strata of tumor‐infiltrating lymphocyte levels
Source: Clin Transl Med. 2022 Nov 27;12(11):e1114. doi: 10.1002/ctm2.1114 (PMC9702366; doi:10.1002/ctm2.1114)
Supplement: Supplementary file 1 — Supporting Information [file CTM2-12-e1114-s001.docx]

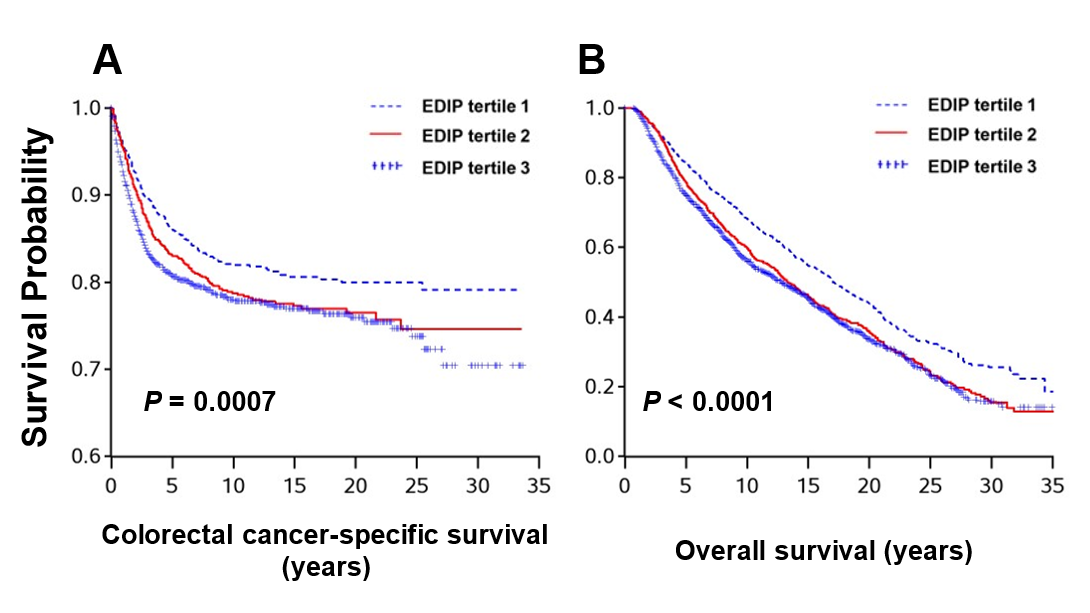


**Supplementary Figure 1**. Kaplan-Meier survival curves of patients with colorectal cancer according to post-diagnosis EDIP scores among all confirmed colorectal cancer patients. The *P* values were calculated using the log-rank test (two-sided). A. Colorectal cancer-specific survival; B. Overall survival.

**Supplementary Table 1**. The distribution of post-diagnosis empirical dietary pattern scores in the Nurses’ Health Study (NHS) and the Health Professionals Follow-up Study (HPFS)

| Percentile | Post-diagnosis empirical dietary pattern (EDIP) score | | |
| --- | --- | --- | --- |
|  | The Nurses’ Health Study |  | The Health Professionals Follow-up Study |
| Minimum | -1.51 |  | -1.73 |
| 1% | -0.77 |  | -0.95 |
| 5% | -0.49 |  | -0.57 |
| 10% | -0.37 |  | -0.41 |
| 25% | -0.18 |  | -0.17 |
| 50% | 0 |  | 0 |
| 75% | 0.14 |  | 0.18 |
| 90% | 0.32 |  | 0.38 |
| 95% | 0.46 |  | 0.55 |
| 99% | 0.77 |  | 0.89 |
| Maximum | 1.82 |  | 1.47 |

**Supplementary Table 2**. Characteristics of colorectal cancer patients with available immune reaction status according to tertiles of post-diagnosis empirical dietary pattern scores in the Nurses’ Health Study (NHS) and the Health Professionals Follow-up Study (HPFS)¶

| Characteristic* | All cases | Post-diagnosis empirical dietary  inflammatory pattern (EDIP) scores | | | *P*-value |
| --- | --- | --- | --- | --- | --- |
|  |  | Tertile 1 (lowest) | Tertile 2 | Tertile 3 (highest) |  |
| Participants (n) | 1192 | 429 | 355 | 408 |  |
| Age at diagnosis, year | 69.6 (8.8) | 69.3 (8.4) | 70.4 (9.0) | 69.1 (9.0) | 0.051 |
| Sex (n, %) |  |  |  |  | 0.44 |
| Female (NHS) | 669 (56) | 249 (58) | 190 (54) | 230 (56) |  |
| Male (HPFS) | 523 (44) | 180 (42) | 165 (46) | 178 (44) |  |
| Year of diagnosis (n, %) |  |  |  |  | 0.14 |
| Prior to 1995 | 381 (32) | 147(34) | 101 (28) | 133 (33) |  |
| 1996-2000 | 333 (28) | 121 (28) | 92 (26) | 120 (29) |  |
| 2001-2014 | 478 (40) | 161 (38) | 162 (46) | 155 (38) |  |
| Family history of colorectal cancer (n, %) | 244 (20) | 86 (20) | 73 (22) | 84 (20) | 0.76 |
| Body mass index, kg/m^2^ | 26.0 (3.9) | 25.5 (3.7) | 25.6 (3.5) | 26.8 (4.1) | <0.0001 |
| Alcohol intake, g/day | 6.8 (9.4) | 9.9 (10.5) | 6.0 (8.6) | 4.3 (7.9) | <0.0001 |
| Pack-year of smoking | 14.7 (17.6) | 16.1 (17.9) | 12.2 (15.9) | 15.4 (18.6) | 0.002 |
| Physical activity, METS - h/week | 15.9 (16.0) | 18.1 (16.4) | 15.7 (15.8) | 13.9 (15.4) | 0.0005 |
| Regular aspirin user (n, %) | 445 (38) | 173 (40) | 126 (39) | 146 (36) | 0.47 |
| Total energy intake, kcal/day | 1805 (571) | 1788 (578) | 1746 (565) | 1876 (561) | 0.027 |
| Tumor location (n, %) |  |  |  |  | 0.78 |
| Proximal colon cancer | 568 (48) | 196 (46) | 170 (48) | 202 (50) |  |
| Distal colon cancer | 357 (30) | 126 (30) | 111 (31) | 120 (29) |  |
| Rectal cancer | 264 (22) | 105 (24) | 73 (21) | 86 (21) |  |
| Tumor differentiation (n, %) |  |  |  |  | 0.45 |
| Well to moderate | 1085 (92) | 390 (92) | 320 (90) | 375 (93) |  |
| Poor | 98 (8) | 34 (8) | 34 (10) | 30 (7) |  |
| AJCC disease stage (n, %) |  |  |  |  | 0.21 |
| I | 330 (28) | 126 (29) | 89 (26) | 115 (28) |  |
| II | 387 (32) | 142 (33) | 127 (37) | 118 (29) |  |
| III | 311 (26) | 106 (25) | 97 (26) | 108 (26) |  |
| IV | 65 (5) | 23 (5) | 12 (3) | 30 (7) |  |
| Unknown | 99 (8) | 32 (7) | 30 (8) | 37 (9) |  |
| MSI status (n, %) |  |  |  |  | 0.019 |
| Non-MSI-high | 866 (82) | 325 (87) | 254 (81) | 287 (79) |  |
| MSI-high | 186 (18) | 50 (13) | 60 (19) | 76 (21) |  |
| CIMP status (n, %) |  |  |  |  | 0.13 |
| CIMP-low/negative | 815 (81) | 300 (84) | 223 (80) | 276 (79) |  |
| CIMP-high | 192 (19) | 56 (16) | 61 (20) | 75 (21) |  |
| *KRAS* mutation (n, %) |  |  |  |  | 0.42 |
| Wild type | 584 (59) | 202 (56) | 182 (61) | 200 (59) |  |
| Mutant | 411 (41) | 158 (44) | 116 (39) | 137 (41) |  |
| *BRAF* mutation (n, %) |  |  |  |  | 0.58 |
| Wild type | 905 (85) | 325 (85) | 276 (87) | 304 (84) |  |
| Mutant | 155 (15) | 55 (15) | 42 (13) | 58 (16) |  |
| *PIK3CA* mutation (n, %) |  |  |  |  | 0.75 |
| Wild type | 822 (83) | 299 (83) | 249 (82) | 274 (84) |  |
| Mutant | 171 (17) | 64 (17) | 55 (18) | 52 (16) |  |
| PTGS2 (cyclooxygenase-2) expression (n, %) |  |  |  |  | 0.24 |
| Negative | 379 (39) | 140 (41) | 119 (42) | 120 (36) |  |
| Positive | 587 (61) | 200 (59) | 170 (59) | 217 (64) |  |
| CD274 (PD-L1) |  |  |  |  | 0.45 |
| Low | 249 (40) | 85 (40) | 85 (42) | 79 (36) |  |
| High | 379 (60) | 125 (60) | 116 (58) | 138 (64) |  |
| LINE-1 methylation level (%) | 63.9 (10.1) | 63.5 (10.2) | 64.0 (10.3) | 64.2 (9.8) | 0.77 |
| Tumor-infiltrating lymphocytes |  |  |  |  | 0.77 |
| Absent/low | 860 (72) | 313 (73) | 258 (73) | 289 (71) |  |
| Intermediate/high | 329 (28) | 115 (27) | 96 (27) | 118 (29) |  |
| Intratumoral periglandular reaction |  |  |  |  | 0.066 |
| Absent/low | 137 (12) | 39 (9) | 40 (11) | 58 (14) |  |
| Intermediate/high | 1052 (88) | 389 (91) | 314 (89) | 349 (86) |  |
|  |  |  |  |  |  |
|  |  |  |  |  |  |
| Peritumoral reaction |  |  |  |  | 0.16 |
| Absent/low | 138 (12) | 42 (10) | 39 (11) | 57 (14) |  |
| Intermediate/high | 1046 (88) | 385 (90) | 312 (89) | 349 (86) |  |
| Crohn’s-like reaction |  |  |  |  | 0.63 |
| Absent/low | 716 (73) | 272 (75) | 203 (71) | 241 (72) |  |
| Intermediate/high | 265 (27) | 92 (25) | 80 (28) | 93 (28) |  |

Abbreviations: AJCC, American Joint Committee on Cancer; CIMP, CpG island methylator phenotype-specific promoters; EDIP, empirical dietary inflammatory pattern; HPFS, Health Professionals Follow-up Study; LINE-1, long interspersed nucleotide element-1; METS, metabolic equivalent task score; MSI, microsatellite instability; NHS, Nurses’ Health Study.

¶ Post-diagnosis EDIP scores were estimated based on the first questionnaire returned between 6 and 48 months after diagnosis of colorectal cancer.

* Continuous variables are shown as mean (standard deviation). Percentage (%) indicates the proportion of cases with a specific clinical, pathological, or molecular characteristic in cases with available immune reaction status according to tertiles of post-diagnosis EDIP scores.

**Supplementary Table 3**. The components of post-diagnosis empirical dietary inflammatory pattern scores and 5-year mortality among all confirmed colorectal cancer patients in the Nurses’ Health Study (NHS) and the Health Professionals Follow-up Study (HPFS)¶

| The components of EDIP score | HR (95% CI) of 5-year colorectal cancer-specific mortality for the components of EDIP score* | | |  | HR (95% CI) of 5-year all-cause mortality for the components of EDIP score* | | |
| --- | --- | --- | --- | --- | --- | --- | --- |
|  | Tertile 1 | Tertile 2 | Tertile 3 |  | Tertile 1 | Tertile 2 | Tertile 3 |
| Coffee | 1 (reference) | 0.76 (0.60-0.98) | 0.72 (0.56-0.92) |  | 1 (reference) | 0.75 (0.62-0.92) | 0.72 (0.58-0.89) |
| Processed meat | 1 (reference) | 1.43 (1.10-1.87) | 1.54 (1.14-2.08) |  | 1 (reference) | 1.41 (1.13-1.76) | 1.45 (1.13-1.86) |
| Red meat | 1 (reference) | 0.84 (0.64-1.09) | 1.08 (0.82-1.43) |  | 1 (reference) | 0.92 (0.74-1.14) | 1.04 (0.82-1.31) |
| Organ meat | 1 (reference) | 0.98 (0.76-1.26) | 0.98 (0.76-1.26) |  | 1 (reference) | 0.94 (0.76-1.17) | 1.09 (0.91-1.31) |
| Fish (other than dark-meat fish) | 1 (reference) | 0.91 (0.71-1.16) | 1.02 (0.78-1.35) |  | 1 (reference) | 0.89 (0.73-1.08) | 0.96 (0.76-1.21) |
| Refined grains | 1 (reference) | 0.99 (0.77-1.27) | 1.18 (0.91-1.52) |  | 1 (reference) | 0.99 (0.81-1.21) | 1.10 (0.89-1.36) |
| Dark-yellow vegetables | 1 (reference) | 0.87 (0.68-1.10) | 0.78 (0.59-1.03) |  | 1 (reference) | 0.92 (0.75-1.14) | 0.89 (0.71-1.12) |
| Green-leafy vegetables | 1 (reference) | 0.98 (0.77-1.25) | 0.85 (0.65-1.12) |  | 1 (reference) | 0.98 (0.80-1.20) | 0.93 (0.74-1.16) |
| Other vegetables | 1 (reference) | 0.91 (0.70-1.17) | 1.06 (0.79-1.41) |  | 1 (reference) | 1.00 (0.81-1.24) | 1.01 (0.79-1.29) |
| Tomatoes | 1 (reference) | 1.23 (0.96-1.57) | 1.25 (0.96-1.62) |  | 1 (reference) | 1.08 (0.88-1.33) | 1.11 (0.89-1.38) |
| Beer | 1 (reference) | 1.33 (0.95-1.87) | 1.30 (0.93-1.81) |  | 1 (reference) | 1.31 (0.99-1.74) | 1.22 (0.92-1.60) |
| Wine | 1 (reference) | 0.90 (0.56-1.44) | 1.07 (0.85-1.35) |  | 1 (reference) | 0.87 (0.59-1.27) | 0.98 (0.81-1.19) |
| Tea | 1 (reference) | 1.24 (0.97-1.59) | 1.13 (0.90-1.42) |  | 1 (reference) | 1.22 (1.00-1.45) | 1.03 (0.85-1.25) |
| Fruit juice | 1 (reference) | 0.87 (0.69-1.11) | 0.98 (0.76-1.27) |  | 1 (reference) | 0.95 (0.78-1.22) | 0.99 (0.80-1.23) |
| High-energy beverages | 1 (reference) | 1.30 (1.01-1.67) | 1.10 (0.87-1.40) |  | 1 (reference) | 1.12 (0.90-1.39) | 1.11 (0.91-1.34) |
| Low-energy beverages | 1 (reference) | 1.04 (0.80-1.36) | 0.98 (0.80-1.36) |  | 1 (reference) | 1.11 (0.89-1.38) | 1.01 (0.84-1.22) |
| Pizza | 1 (reference) | 0.96 (0.71-1.29) | 0.89 (0.70-1.13) |  | 1 (reference) | 0.81 (0.63-1.04) | 0.86 (0.70-1.04) |
| Snacks | 1 (reference) | 0.80 (0.62-1.03) | 0.96 (0.74-1.25) |  | 1 (reference) | 0.91 (0.74-1.12) | 0.98 (0.78-1.16) |

Abbreviations: CI, confidence interval; EDIP, empirical dietary inflammatory pattern; HR, hazard ratio.

¶ The components of post-diagnosis EDIP scores were estimated based on the first questionnaire returned between 6 and 48 months after diagnosis of colorectal cancer.

* The inverse probability weighting method (for post-diagnosis questionnaire data availability) was integrated into the Cox proportional hazards regression models. All Cox regression models were stratified by sex and tumor stage, and adjusted for age at diagnosis, year of diagnosis, tumor differentiation, tumor location, family history of colorectal cancer, pre-diagnosis empirical dietary pattern scores, post-diagnosis aspirin use, post-diagnosis pack-years of smoking, post-diagnosis alcohol use, post-diagnosis physical activity, post-diagnosis body mass index, and post-diagnosis total energy intake. In addition, the components of EDIP were also mutually adjusted.

**Supplementary Table 4**. Post-diagnosis empirical dietary inflammatory pattern scores and mortality stratified by body mass index in the Nurses’ Health Study (NHS) and the Health Professionals Follow-up Study (HPFS)¶

| Post-diagnosis  EDIP scores |  | 5-year colorectal cancer-specific mortality | | |  | 5-year all-cause mortality | | |
| --- | --- | --- | --- | --- | --- | --- | --- | --- |
|  | No. of cases | No. of events | Age-adjusted  HR* (95% CI) | Multivariable HR*† (95% CI) |  | No. of events | Age-adjusted  HR* (95% CI) | Multivariable  HR*† (95% CI) |
| **BMI <25** |  |  |  |  |  |  |  |  |
| Tertile 1 | 476 | 62 | 1 (reference) | 1 (reference) |  | 83 | 1 (reference) | 1 (reference) |
| Tertile 2 | 396 | 57 | 1.08  (0.78-1.50) | 0.99  (0.71-1.37) |  | 86 | 1.16  (0.88-1.54) | 1.04  (0.79-1.38) |
| Tertile 3 | 375 | 70 | 1.30  (0.95-1.76) | 1.10  (0.80-1.51) |  | 109 | 1.53  (1.17-2.00) | 1.29  (0.99-1.69) |
| *P*_trend_ |  |  | 0.10 | 0.57 |  |  | 0.002 | 0.057 |
| **BMI ≥25** |  |  |  |  |  |  |  |  |
| Tertile 1 | 449 | 31 | 1 (reference) | 1 (reference) |  | 50 | 1 (reference) | 1 (reference) |
| Tertile 2 | 506 | 54 | 1.29  (0.87-1.93) | 1.45  (0.95-2.21) |  | 87 | 1.32  (0.095-1.82) | 1.25  (0.90-1.73) |
| Tertile 3 | 537 | 65 | 1.63  (1.11-2.40) | 2.13  (1.34-3.38) |  | 99 | 1.58  (1.16-2.16) | 1.53  (1.12-2.10) |
| *P*_trend_ |  |  | 0.012 | 0.001 |  |  | 0.004 | 0.007 |
| *P*_interaction_‡ |  |  | 0.23 | 0.066 |  |  | 0.98 | 0.47 |

Abbreviations: CI, confidence interval; EDIP, empirical dietary inflammatory pattern; HR, hazard ratio.

¶ Post-diagnosis EDIP scores were estimated based on the first questionnaire returned between 6 and 48 months after diagnosis of colorectal cancer.

* The inverse probability weighting method (for post-diagnosis questionnaire data availability) was integrated into the Cox proportional hazards regression models. All Cox regression models were stratified by sex and tumor stage, and adjusted for age at diagnosis.

† Multivariable Cox regression models originally included the following variables: year of diagnosis, tumor differentiation, tumor location, family history of colorectal cancer, pre-diagnosis empirical dietary pattern scores, post-diagnosis aspirin use, post-diagnosis pack-years of smoking, post-diagnosis physical activity, post-diagnosis total energy intake, post-diagnosis body mass index, and post-diagnosis alcohol use if these factors are not as stratification factors. A backward stepwise selection was used to select the variables for the final models.

‡ *P*_interaction_ (two-sided) was calculated by the Wald test for the cross-product of post-diagnosis EDIP scores (ordinal) and body mass index (<25 vs. ≥25) in the Cox regression model.

**Supplementary Table 5**. Post-diagnosis empirical dietary inflammatory pattern scores and mortality stratified by stage in the Nurses’ Health Study (NHS) and the Health Professionals Follow-up Study (HPFS)¶

| Post-diagnosis  EDIP scores |  | 5-year colorectal cancer-specific mortality | | |  | 5-year all-cause mortality | | |
| --- | --- | --- | --- | --- | --- | --- | --- | --- |
|  | No. of cases | No. of events | Age-adjusted  HR* (95% CI) | Multivariable HR*† (95% CI) |  | No. of events | Age-adjusted  HR* (95% CI) | Multivariable  HR*† (95% CI) |
| **Stage I-III** |  |  |  |  |  |  |  |  |
| Tertile 1 | 729 | 57 | 1 (reference) | 1 (reference) |  | 93 | 1 (reference) | 1 (reference) |
| Tertile 2 | 645 | 66 | 1.26  (0.89-1.79) | 1.23  (0.86-1.74) |  | 106 | 1.20  (0.91-1.57) | 1.15  (0.87-1.52) |
| Tertile 3 | 653 | 77 | 1.49  (1.07-2.09) | 1.45  (1.03-2.03) |  | 137 | 1.63  (1.26-2.11) | 1.57  (1.21-2.04) |
| *P*_trend_ |  |  | 0.03 | 0.04 |  |  | 0.004 | 0.007 |
| **Stage IV** |  |  |  |  |  |  |  |  |
| Tertile 1 | 51 | 33 | 1 (reference) | 1 (reference) |  | 33 | 1 (reference) | 1 (reference) |
| Tertile 2 | 59 | 41 | 1.09  (0.75-1.59) | 1.38  (0.91-2.10) |  | 43 | 1.13  (0.78-1.64) | 1.30  (0.87-1.96) |
| Tertile 3 | 76 | 54 | 1.15  (0.80-1.63) | 1.63  (1.10-2.42) |  | 56 | 1.19  (0.84-1.70) | 1.63  (1.11-2.40) |
| *P*_trend_ |  |  | 0.53 | 0.03 |  |  | 0.32 | 0.008 |
| *P*_interaction_‡ |  |  | 0.40 | 0.33 |  |  | 0.55 | 0.60 |

Abbreviations: CI, confidence interval; EDIP, empirical dietary inflammatory pattern; HR, hazard ratio.

¶ Post-diagnosis EDIP scores were estimated based on the first questionnaire returned between 6 and 48 months after diagnosis of colorectal cancer.

* The inverse probability weighting method (for post-diagnosis questionnaire availability) was integrated into the Cox proportional hazards regression models. All Cox regression models were stratified by sex and tumor stage, and adjusted for age at diagnosis.

† Multivariable Cox regression models originally included the following variables: year of diagnosis, tumor differentiation, tumor location, family history of colorectal cancer, pre-diagnosis empirical dietary pattern scores, post-diagnosis aspirin use, post-diagnosis pack-years of smoking, post-diagnosis alcohol use, post-diagnosis physical activity, post-diagnosis body mass index, and post-diagnosis total energy intake. A backward stepwise selection was used to select the variables for the final models.

‡ *P*_interaction_ (two-sided) was calculated by the Wald test for the cross-product of post-diagnosis EDIP scores (ordinal) and stage (I-III vs. IV) in the Cox regression model.

**Supplementary Table 6**. Post-diagnosis empirical dietary inflammatory pattern scores and mortality among all confirmed colorectal cancer patients in the Nurses’ Health Study (NHS) and the Health Professionals Follow-up Study (HPFS)¶

| Post-diagnosis  EDIP scores |  | Colorectal cancer-specific mortality | | |  | All-cause mortality | | |
| --- | --- | --- | --- | --- | --- | --- | --- | --- |
|  | No. of cases | No. of events | Age-adjusted  HR* (95% CI) | Multivariable HR*† (95% CI) |  | No. of events | Age-adjusted  HR* (95% CI) | Multivariable  HR*† (95% CI) |
| Tertile 1 | 946 | 171 | 1 (reference) | 1 (reference) |  | 557 | 1 (reference) | 1 (reference) |
| Tertile 2 | 936 | 194 | 1.08 (0.89-1.31) | 1.09 (0.90-1.32) |  | 612 | 1.14 (1.02-1.27) | 1.13 (1.01-1.26) |
| Tertile 3 | 947 | 208 | 1.21 (1.00-1.46) | 1.14 (0.95-1.39) |  | 660 | 1.30 (1.17-1.45) | 1.24 (1.10-1.38) |
| *P*_trend_** |  |  | 0.04 | 0.16 |  |  | <0.0001 | <0.0001 |

Abbreviations: CI, confidence interval; EDIP, empirical dietary inflammatory pattern; HR, hazard ratio.

¶ Post-diagnosis EDIP scores were estimated based on the first questionnaires returned between 6 and 48 months after diagnosis of colorectal cancer.

* The inverse probability weighting method (for post-diagnosis questionnaire data availability) was integrated into the Cox proportional hazards regression models. All Cox regression models were stratified by sex and tumor stage, and adjusted for age at diagnosis.

† Multivariable Cox regression models originally included the following variables: year of diagnosis, tumor differentiation, tumor location, family history of colorectal cancer, pre-diagnosis empirical dietary pattern scores, post-diagnosis aspirin use, post-diagnosis pack-years of smoking, post-diagnosis alcohol use, post-diagnosis physical activity, post-diagnosis body mass index, and post-diagnosis total energy intake. A backward stepwise selection was used to select the variables for the final models.

^**^ *P*_trend_ was calculated using the EDIP score as a continuous variable with the cohort-specific ceilings of the 5th and 95th percentiles.

**Supplementary Table 7**. Empirical dietary inflammatory pattern scores derived from the first questionnaire returned between 12 and 48 months after diagnosis and mortality among all confirmed colorectal cancer patients in the Nurses’ Health Study (NHS) and the Health Professionals Follow-up Study (HPFS)¶

| Post-diagnosis  EDIP scores |  | 5-year colorectal cancer-specific mortality | | |  | 5-year all-cause mortality | | |
| --- | --- | --- | --- | --- | --- | --- | --- | --- |
|  | No. of cases | No. of events | Age-adjusted  HR* (95% CI) | Multivariable HR*† (95% CI) |  | No. of events | Age-adjusted  HR* (95% CI) | Multivariable  HR*† (95% CI) |
| Tertile 1 | 906 | 70 | 1 (reference) | 1 (reference) |  | 111 | 1 (reference) | 1 (reference) |
| Tertile 2 | 901 | 108 | 1.33  (1.02-1.75) | 1.20  (0.91-1.60) |  | 170 | 1.35  (1.08-1.68) | 1.12  (0.89-1.41) |
| Tertile 3 | 896 | 118 | 1.46  (1.12-1.90) | 1.43  (1.10-1.87) |  | 190 | 1.55  (1.25-1.92) | 1.45  (1.16-1.80) |
| *P*_trend_^**^ |  |  | 0.04 | 0.05 |  |  | 0.003 | 0.001 |

Abbreviations: CI, confidence interval; EDIP, empirical dietary inflammatory pattern; HR, hazard ratio.

¶ Post-diagnosis EDIP scores were estimated based on the first questionnaire returned between 12 and 48 months after diagnosis of colorectal cancer.

* The inverse probability weighting method (for post-diagnosis questionnaire data availability) was integrated into the Cox proportional hazards regression models. All Cox regression models were stratified by sex and tumor stage, and adjusted for age at diagnosis.

† Multivariable Cox regression models originally included the following variables: year of diagnosis, tumor differentiation, tumor location, family history of colorectal cancer, pre-diagnosis empirical dietary pattern scores, post-diagnosis aspirin use, post-diagnosis pack-years of smoking, post-diagnosis alcohol use, post-diagnosis physical activity, post-diagnosis body mass index, and post-diagnosis total energy intake. A backward stepwise selection was used to select the variables for the final models.

^**^ *P*_trend_ was calculated using EDIP score as a continuous variable with the cohort-specific ceilings of the 5th and 95th percentiles of the score.

**Supplementary Table 8**. Post-diagnosis empirical dietary inflammatory pattern scores and mortality after adjusting for time between cancer diagnosis and the timing of first questionnaire returned ¶

| Post-diagnosis  EDIP scores |  | 5-year colorectal cancer-specific mortality | | |  | 5-year all-cause mortality | | |
| --- | --- | --- | --- | --- | --- | --- | --- | --- |
|  | No. of cases | No. of events | Age-adjusted  HR* (95% CI) | Multivariable HR*† (95% CI) |  | No. of events | Age-adjusted  HR* (95% CI) | Multivariable  HR*† (95% CI) |
| Tertile 1 | 914 | 112 | 1 (reference) | 1 (reference) |  | 161 | 1 (reference) | 1 (reference) |
| Tertile 2 | 860 | 118 | 1.14 (0.90-1.44) | 1.14 (0.90-1.45) |  | 185 | 1.22 (1.00-1.49) | 1.18 (0.97-1.44) |
| Tertile 3 | 889 | 140 | 1.45 (1.15-1.81) | 1.41 (1.12-1.77) |  | 209 | 1.52 (1.25-1.84) | 1.44 (1.19-1.74) |
| *P*_trend_** |  |  | 0.002 | 0.003 |  |  | <0.0001 | 0.0004 |

Abbreviations: CI, confidence interval; EDIP, empirical dietary inflammatory pattern; HR, hazard ratio.

¶ Post-diagnosis EDIP scores were estimated based on the first questionnaires returned between 6 and 48 months after diagnosis of colorectal cancer.

* The inverse probability weighting method (for post-diagnosis questionnaire data availability) was integrated into the Cox proportional hazards regression models. All Cox regression models were stratified by sex and tumor stage and adjusted for age at diagnosis and time from cancer diagnosis to the timing of post-diagnosis EDIP assessment after cancer diagnosis.

† Multivariable Cox regression models originally included the following variables: year of diagnosis, tumor differentiation, tumor location, family history of colorectal cancer, pre-diagnosis empirical dietary pattern scores, post-diagnosis aspirin use, post-diagnosis pack-years of smoking, post-diagnosis alcohol use, post-diagnosis physical activity, post-diagnosis body mass index, and post-diagnosis total energy intake. A backward stepwise selection was used to select the variables for the final models.

^**^ *P*_trend_ was calculated using the EDIP score as a continuous variable with the cohort-specific ceilings of the 5th and 95th percentiles.
